# Supplementary material for: How chronic conditions are understood, experienced and managed within African communities in Europe, North America and Australia: A synthesis of qualitative studies
Source: PLoS One. 2023 Feb 15;18(2):e0277325. doi: 10.1371/journal.pone.0277325 (PMC9931108; doi:10.1371/journal.pone.0277325)
Supplement: S2 Table — (DOCX) [file pone.0277325.s002.docx]

**S2 Table. Search Strategy**

| **Number** | **Search terms** |
| --- | --- |
| **# S1** | Chronic Conditions OR Chronic Diseases, Chronic NCDs OR Chronic Non-Communicable Diseases OR NCDs OR Non-Communicable Diseases OR Chronicity OR Chronic Illness OR Chronic Conditions OR Diabetes OR Cancers OR Stroke OR Hypertension OR Coronary heart diseases OR Neurodegenerative Disorders OR Dementia OR Alzheimer OR Mental Health OR Sickle Cell OR Cardiovascular disease OR Parkinson OR Asthma OR Huntington OR Depression OR Anxiety OR Chronic Stress OR Emotional Stress OR Overweight OR Obesity OR Heart Attacks OR Heart Failure OR Kidney Disease OR Diabetes Mellitus OR Chronic Lung Diseases OR Ischaemic Heart Disease OR Chronic Respiratory Diseases OR Myocardial Infarction OR Coronary Heart Disease OR High Blood Pressure OR Chronic Obstructive Pulmonary Disease |
| **# S2** | Lived experiences OR Access to care OR Care OR Accessing care OR Experiences OR Coping OR Expressions OR Health Seeking Behaviour OR Care Seeking OR Attitude to Health OR Ethnicity OR Health Beliefs OR Explanatory Models OR Attitude to Health |
| **# S3** | Interviews OR Qualitative Interviews OR Focus Group Discussions OR Focus Group OR Participant observation OR Ethnography OR Mixed Methods OR Accounts OR Unstructured interviews OR Phenomenology OR Grounded Theory OR Case Study OR Document Analysis OR Narrative Interviews OR Narratives OR Photovoice OR Qualitative study OR Qualitative inquiry OR Qualitative Analysis |
| **#S4** | African Diaspora OR African Refugee OR African Immigrant OR Black African OR African Ethnic Minorities OR Diasporas OR Racial Minority Groups OR Ethnic Minority Groups OR African Diasporas OR African Europeans OR Black African Background OR Black British OR African-Caribbean OR Afro-Caribbean OR Anglo-African OR Undocumented Immigrants OR African Minority Group OR sub-Saharan African descent OR Africa OR Africans OR African OR African Surinamese OR African Continental Ancestry Group OR African Descent OR African Ancestry OR Minority Groups OR Ethnic Minority Groups OR Underrepresented OR African Community OR African Communities |
| **#S5** | Austria OR Belgium OR Denmark OR Finland OR France OR Germany OR Iceland OR Ireland OR Italy OR Netherlands OR Norway OR Poland OR Portugal OR Spain OR Sweden OR Switzerland OR United Kingdom OR England OR Wales OR UK OR Vatican City OR Holy See OR Scotland OR Northern Ireland OR Ireland OR Australia OR New Zealand OR North America OR Canada OR US OR USA OR United States of America OR Global North OR Developed Countries OR High Income Countries OR Europe OR EU OR European Region OR European Union |
| **#S6** | S1 AND S2 AND S3 |
| **#S7** | S4 OR S5 |
| **#S9** | S6 AND S7 |
| **Limiters** | Human, English Language, Full Text |
