# Supplementary material for: How chronic conditions are understood, experienced and managed within African communities in Europe, North America and Australia: A synthesis of qualitative studies
Source: PLoS One. 2023 Feb 15;18(2):e0277325. doi: 10.1371/journal.pone.0277325 (PMC9931108; doi:10.1371/journal.pone.0277325)
Supplement: S3 Table — (DOCX) [file pone.0277325.s003.docx]

**S2 Table. COREQ-32 Checklist**

|  | **Checklist** | **[16]**  **Allard et al, 2018** | **[17]**  **Alloh et al, 2018** | **[18]**  **Alloh et al., 2021** | **[19]**  **Anderson, et al 2013** | **[20]**  **Asgary, 2011** | **[21]**  **Bamidele et al 2019** |
| --- | --- | --- | --- | --- | --- | --- | --- |
| 1 | **Interviewer/facilitator** | NA | Not stated | Not stated | Not stated | Not stated | OB, HM, BML, and EM |
| 2 | **Credentials** | Not stated | Not stated | Not stated | Not stated | Yes | Not stated |
| 3 | **Occupation** | General practitioner | Not stated | Not stated | Yes | Not stated | Not stated |
| 4 | **Gender** | Not stated | Not stated | Female | Female | Not stated | Male and Female |
| 5 | **Experience and training** | Experienced in using interpreters and trained in qualitative research techniques | Not stated | Not stated | Yes | Not stated | Not stated |
| 6 | **Relationship established** | No | No | Not stated | Not stated | Not stated | Yes |
| 7 | **Participant knowledge of the interviewer** | No | Not stated | Not stated | Not stated | Not stated | No |
| 8 | **Interviewer characteristics** | Not stated | Not stated | Not stated | Not stated | Not stated | Not stated |
| 9 | **Methodological orientation and Theory** | No | Constructivist Grounded Theory | Constructive Grounded Theory | Exploratory qualitative study and phenomenological approach | Not stated | Not stated |
| 10 | **Sampling** | Not stated | Purposive and snow balling | Not stated | Purposive sampling | Purposive sampling | Convenience and snowball sampling |
| 11 | **Method of approach** | Face-to-face | Face-to-face | Face-to-face | Face-to-face | Face-to-face | Face-to-face |
| 12 | **Sample size** | 4 | 34 | 34 | 7 | 35 | 47 |
| 13 | **Non-participation** | Not stated | 16 | 16 | 23 | Not stated | None |
| 14 | **Setting of data collection** | Hospital and community | Community, support groups; churches; mosques | Community, support groups; churches; mosques | Healthcare | 2 clinics | Mixed, home, work, HCF |
| 15 | **Presence of nonparticipants** | Not stated | No | No | No | Not stated | No |
| 16 | **Description of sample** | N: 19  Gender: 8m, 11w  Age: 18 - 64  Location: Victoria  Setting: hospital; 3 community clinics | N: 34  Gender: 15m,19m  Age: 33-82  Location: London  Setting: Community, support groups; churches; mosques | N: 34  Gender: 15m,19m  Age: 33-82  Location: London  Setting: Community, support groups; churches; mosques | N= *3 African men* (Total – 7)  Gender: men  Age: 60 -76  Location: South England  Setting: Healthcare | N: 35  Gender: 30m; 5w  Age: under 40  Location: New York  Setting: 2 clinics | N: 47  Gender: 25men  Age: 65 yrs or less  Location: England  Setting: Mixed, home, work, HCF |
| 17 | **Interview guide** | Yes | Yes | Yes | Yes | No, not provided; pilot testing not mentioned. | Yes |
| 18 | **Repeat interviews** | No | No | No | Not stated | No, not stated | Not stated |
| 19 | **Audio/visual recording** | No | Yes | Yes | Yes | Yes (audio-taped) | Yes |
| 20 | **Field Notes** | Field notes were provided | Field notes were provided | Field notes were provided | Field notes were provided | Field notes were provided | Not stated |
| 21 | **Duration** | Not stated | 60 minutes | 60 minutes | 60 minutes | Not stated | 30 to 90 minutes |
| 22 | **Data saturation** | Not stated | Yes | Yes | Not stated | Not stated | Yes |
| 23 | **Transcripts returned** | No | No | No | Not stated | Not stated | Not stated |
| 24 | **Number of data coders** | Not stated | 1 | 1 | Not stated | Not stated | Four |
| 25 | **Description of the coding tree** | No | Yes | Yes | Yes | Not stated | Yes |
| 26 | **Derivation of themes** | Yes | Yes | Yes | Yes | Themes derived from data | Yes |
| 27 | **Software** | Not stated | Nvivo 11 by QSR International | Nvivo 11 by QSR International | Not stated | Not stated | NVivo 11 |
| 28 | **Participant checking** | No | No | No | Not stated | Not stated | Not stated |
| 29 | **Quotations presented** | Yes | Yes | Yes | Yes | Yes | Yes |
| 30 | **Data and findings consistent** | Yes | Yes | Yes | Yes | Yes | Yes |
| 31 | **Clarity of major themes** | Yes | Yes | Yes | Yes | Yes | Yes |
| 32 | **Clarity of minor themes** | No | No | No | Yes | Yes | Yes |

|  | **Checklist** | **[22]**  **Bettmann, et al 2015** | **[23]**  **Beune et al, 2006** | **[24]**  **Beune et al, 2008** | **[25]**  **Cooper et al 2012** | **[26]**  **Hjelm et al 2018** | **[27]**  **Hjelm et al 2012** |
| --- | --- | --- | --- | --- | --- | --- | --- |
| 1 | **Interviewer/facilitator** | Not stated | Not stated | Not stated | Not stated | Not stated | Not stated |
| 2 | **Credentials** | Yes | Not stated | Not stated | Yes | Yes | Yes |
| 3 | **Occupation** | Not stated | Not stated | Not stated | Yes | Yes | Yes |
| 4 | **Gender** | Not stated | Male | Male | Male and Female | Yes | Yes |
| 5 | **Experience and training** | Yes | Not stated | Not stated | Yes | Not stated | Not stated |
| 6 | **Relationship established** | Yes | Not stated | Not stated | Not stated | Not stated | Not stated |
| 7 | **Participant knowledge of the interviewer** | No | Not stated | Not stated | Not stated | Not stated | Not stated |
| 8 | **Interviewer characteristics** | Graduate-level research assistants | Not stated | Not stated | Not stated | Not stated | Not stated |
| 9 | **Methodological orientation and Theory** | No | Thematic analysis | Thematic analysis | Purnell’s (2002) model of cultural competence | Content analysis | Content analysis |
| 10 | **Sampling** | Purposive and snowball sampling | Purposive | Purposive | Purposive sampling | Convenience sampling | Consecutive sampling |
| 11 | **Method of approach** | Face-to-face | Face-to-face | Face-to-face | Face-to-face | Face-to-face | Face-to-face |
| 12 | **Sample size** | 20 | 46 | 46 | 14 | 9 | 23 |
| 13 | **Non-participation** | No | 11 | 11 | None | Not stated | Not stated |
| 14 | **Setting of data collection** | Community | Primary care centres | primary care centres | community organisations, 2 protestant churches, 5 ‘African societies based on language or country of origin’ | clinic; homes | Clinic |
| 15 | **Presence of nonparticipants** | No | Not stated | Not stated | No | Not stated | Not stated |
| 16 | **Description of sample** | N: 20  Gender: 10m. 10w  Age: n/a  Location: Salt lake City  Setting: community | N: *19* (total 54)  Gender: 20m, 26w  Age: 35-65yrs  Location: Amsterdam  Setting: Primary care centres | N:*19* (total 54)  Gender: 20m, 26w  Age: 35-65  Location: Amsterdam  Setting: primary care centres | N: 19  Gender:10m,19w  Age: 18-60  Location: Glasgow  Setting: community organisations, 2 protestant churches, 5 ‘African societies based on language or country of origin’ | N: *10* (total 23)  Gender: 10 w  Age: 23-41  Location: Sweden  Setting: clinic; homes | N: *9* (total 23)  Gender: 9w  Age: 23 - 40  Location: Sweden  Setting: clinic |
| 17 | **Interview guide** | Yes | Yes | Yes | Yes | Yes | Yes |
| 18 | **Repeat interviews** | Not conducted | Not stated | Not stated | Not stated | Yes, 3 repeats | Not stated |
| 19 | **Audio/visual recording** | Yes | Yes | Yes | Yes | Yes | Yes |
| 20 | **Field Nots** | Field notes were not provided | Not stated | Not stated | Field notes were provided | Field notes were provided | Field notes were provided |
| 21 | **Duration** | No | 90 Mins | 90Mins | Not stated | 1.5 hours | 1.5 hours |
| 22 | **Data saturation** | Not | Yes | Yes | Yes | Yes | Yes |
| 23 | **Transcripts returned** | No | Not stated | Not stated | Not stated | Not stated | Not stated |
| 24 | **Number of data coders** | 2 | 3 | 2 | Two | 2 | Not stated |
| 25 | **Description of the coding tree** | Yes | Yes | Yes | Yes | Yes | Yes |
| 26 | **Derivation of themes** | Yes | Themes were derived from data | Themes were derived from data | Yes | Themes were derived a priori and from data | Themes were derived a priori and from data |
| 27 | **Software** | No | Maxqda software | Maxqda software | Not stated | Not stated | Not stated |
| 28 | **Participant checking** | No | Not stated | Not stated | Not stated | Not stated | Not stated |
| 29 | **Quotations presented** | Yes | Yes | Yes | Yes | Yes | Yes |
| 30 | **Data and findings consistent** | Yes | Yes | Yes | Yes | Yes | Yes |
| 31 | **Clarity of major themes** | Yes | Yes | Yes | Yes | Yes | Yes |
| 32 | **Clarity of minor themes** | No | Yes | Yes | Yes | Not stated | Not stated |

|  | **Checklist** | **[28]**  **Jager et al 2018** | **[29]**  **Kindarara, et al 2017** | **[30]**  **Kohinor, et al 2011** | **[31]**  **Kohinor et al, 2011** | **[32]**  **Kokanovic et al,**  **2008** | **[33]**  **Maxwell, et al, 1999** |
| --- | --- | --- | --- | --- | --- | --- | --- |
| 1 | **Interviewer/facilitator** | Not stated | Not stated | MJEK | MJEK | Trained research assistants who were  Somali, Amharic and Tigrinya speaking | Not stated |
| 2 | **Credentials** | Not stated | Yes | Not stated | Not stated | Not stated | Not stated |
| 3 | **Occupation** | Not stated | Yes | Not stated | Not stated | Not stated | Not stated |
| 4 | **Gender** | Not clear | Not clear | Male/Female | Male/Female | Male & Female | Male |
| 5 | **Experience and training** | Not stated | Not stated | Not stated | Not stated | Research assistants were trained in qualitative research | Not stated |
| 6 | **Relationship established** | Not stated | Not stated | Not stated | Not stated | No | Not stated |
| 7 | **Participant knowledge of the interviewer** | Not stated | Not stated | Not stated | Not stated | Not stated | Not stated |
| 8 | **Interviewer characteristics** | Not stated | Not stated | Not stated | Not stated | Yes | Not stated |
| 9 | **Methodological orientation and Theory** | Thematic analysis | Content analysis | Grounded theory | grounded theory | Iterative/thematic analysis | Thematic analysis |
| 10 | **Sampling** | purposive sampling | Purposive and snowball sampling | Purposive | Purposive | Participants were 62 women and men, recruited through general practices | Systematic non-random sampling |
| 11 | **Method of approach** | Face-to-face | Face-to-face | Face-to-face | Face-to-face | Face-to-face | Face-to-face |
| 12 | **Sample size** | 12 | 10 | 32 | 32 | 62 women and men | 57 |
| 13 | **Non-participation** | Not stated | Not stated | 11 | 11 | Not stated | Not stated |
| 14 | **Setting of data collection** | dietetic practices | Churches | family general practices | family general practices | Community | Hospital |
| 15 | **Presence of nonparticipants** | Not stated | Not stated | Not stated | Not stated | Not stated | Not stated |
| 16 | **Description of sample** | Y N: *2 African women* (total 12)  Gender: 2w (4m,8w)  Age: 69, 87 (44-87)  Location: Arnhem and Nijmegen regions, Netherlands  Setting: dietetic practices | N: 10  Gender: 5m, 5w  Age: 44 - 76  Location: Sacra-mento county  Setting: churches | N: 16 African Surinamese (total 32)  Gender:12m, 20w  Age: 36-70  Location: Amsterdam  Setting: family general practices | N: 16 African Surinamese (total 32)  Gender: 12m, 20w  Age: 36-70  Location: Amsterdam  Setting: family general practices | N: 62  Gender: NS  Age: NS  Location: Perth  Setting: community | N: *29 West Africans* (Total 57)  Gender: 25m, 32w  Age: 20-60  Location: London  Setting: hospital |
| 17 | **Interview guide** | Yes | Yes | Yes | Yes | Yes | Yes |
| 18 | **Repeat interviews** | Not stated | Not stated | Not stated | Not stated | Not conducted | Not stated |
| 19 | **Audio/visual recording** | Yes | Yes | Yes | Yes | Yes | Yes |
| 20 | **Field Nots** | Not stated | Field notes were not provided | Not stated | Not stated | Field notes were not provided | Not stated |
| 21 | **Duration** | Not stated | 60 to 90 minutes | 60 Mins | 60Mins | 1-2 hours | 90-150Mins |
| 22 | **Data saturation** | Yes | Yes | Yes | Yes | Not stated | Not stated |
| 23 | **Transcripts returned** | Not stated | No | Not stated | Not stated | No | Not stated |
| 24 | **Number of data coders** | 2 | 1 | 3 | 3 | Not stated | Not stated |
| 25 | **Description of the coding tree** | Yes | Yes | Yes | Yes | Yes | Yes |
| 26 | **Derivation of themes** | Themes were derived a priori and from data | Themes were derived a priori | Themes were derived from data | Themes were derived from data | Yes | Themes derived from data |
| 27 | **Software** | Atlas.ti. | Not stated | Maxqda software | MAXQDA software. | No | Nudist version 4 |
| 28 | **Participant checking** | Not stated | Yes | Not stated | Not stated | No | Not stated |
| 29 | **Quotations presented** | Yes | Yes | Yes | Yes | Yes | Yes |
| 30 | **Data and findings consistent** | Yes | Yes | Yes | Yes | Yes | Yes |
| 31 | **Clarity of major themes** | Yes | Yes | Yes | Yes | Yes | Yes |
| 32 | **Clarity of minor themes** | Yes | Yes | Yes | Yes | Yes | Yes |

|  | **Checklist** | **[34]**  **Melamed et al 2019** | **[35]**  **Memon et al, 2016** | **[36]**  **Michaëlis et al, 2015** | **[37]**  **Michlig et al., 2022** | **[38]**  **Mude et al., 2019** | **[39]**  **Mude et al., 2022** |
| --- | --- | --- | --- | --- | --- | --- | --- |
| 1 | **Interviewer/facilitator identified** | Not stated | Not stated | Not stated | Interviews were conducted by Somali facilitators who were trained in research ethics, study design and qualitative data collection techniques. | WB | WB |
| 2 | **Credentials** | Not stated | Not stated | Not stated | PhD | GradDip Pharm, MPH, PhD candidate | GradDip Pharm, MPH, PhD candidate |
| 3 | **Occupation** | Not stated | Not stated | Yes | Not stated | Public Health Officer and PhD candidate | Public Health Officer and PhD candidate |
| 4 | **Gender** | Not stated | Not stated | Male/Female | Female | Male | Male |
| 5 | **Experience and training** | Not stated | Not reported | Not stated | Not stated | Completed an advanced training course in qualitative health research and 10 years of working with refugees in Public Health and  Community Health Promotion services. | Completed an advanced training course in qualitative health research and 10 years of working with refugees in Public Health and  Community Health Promotion services. |
| 6 | **Relationship established** | Not stated | Not reported | Yes | Not stated | Yes | Yes |
| 7 | **Participant knowledge of the interviewer** | Not stated | Not stated | Yes | Not stated | The researcher introduced himself as a PhD candidate from La Trobe University. It is possible that participants also  knew the researcher is a Public Health Officer. | the researcher introduced himself as a PhD candidate from La Trobe University. It is possible that participants also  knew the researcher is a Public Health Officer. |
| 8 | **Interviewer characteristics** | Not stated | Not stated | Yes | Interviewers were trained Somali facilitators | The interviewer is an experienced facilitator with several years of experience as a Public Health Officer and has conducted  several community engagement sessions and research interviews. The interviewer worked as a Public Health Officer with people of diverse backgrounds  including South Sudanese refugees. The interviewer has an interest in health equity and healthcare system access. | The interviewer is an experienced facilitator with several years of experience as a Public Health Officer and has conducted  several community engagement sessions and research interviews. The interviewer worked as a Public Health Officer with people of diverse backgrounds  including South Sudanese refugees. The interviewer has an interest in health equity and healthcare system access. |
| 9 | **Methodological orientation and Theory** | Framework method analysis | Thematic analysis | Content analysis | Social discourse | Interpretive Phenomenology. | Interpretive Phenomenology. |
| 10 | **Sampling** | Not stated | Not stated | Purposive sampling | Purposive | Purposive sampling. | Purposive sampling. |
| 11 | **Method of approach** | Face-to-face | Face-to-face | Face-to-face | Face-to-face | Interested participants were invited through promotional flyers and word-of-mouth to contact the researcher. Participants who  contacted the researcher were screened against specific inclusion criteria, provided with further information about the study and scheduled for an interview. | Interested participants were invited through promotional flyers and word-of-mouth to contact the researcher. Participants who  contacted the researcher were screened against specific inclusion criteria, provided with further information about the study and scheduled for an interview. |
| 12 | **Sample size** | 10 | 26 | 13 | 168 | 15 | 15 |
| 13 | **Non-participation** | 4 | Not stated | 2 | Not stated | None | None |
| 14 | **Setting of data collection** | Onsite at a refugee home and nonclinical setting | community centre | OP services | Community | Community | Community |
| 15 | **Presence of nonparticipants** | Not stated | Not stated | Not stated | Not stated | None | None |
| 16 | **Description of sample** | N: 10  Gender: 10m  Age: 20 - 35  Location: Basel  Setting: Onsite at a refugee home and nonclinical setting | N: *6 Black/Black British* (total – 26)  Gender: 13m, 13w  Age: 18+  Location: Brighton Southeast England  Setting: community centre | N: *2 African women* (total 13)  Gender: 13w  Age: 50 – 60 (33 – 63)  Location: Copenhagen  Setting: OP services | N=168  Gender: m-84; w-84  Age: 14+  Location: Phoenix, Tucson.  Setting: community | N=15  Gender: 8w; 7m  Age: 18-65  Location: Adelaide  Setting: community | N=15  Gender: 8w; 7m  Age: 18-65  Location: Adelaide  Setting: community |
| 17 | **Interview guide** | Yes | Yes | Yes | Interview guide was provided | An interview guide facilitated all the face-to-face interviews. A pilot interview was conducted with two volunteers to test the interview  guide for its ease of understanding, cultural appropriateness and ability to capture the relevant information. | An interview guide facilitated all the face-to-face interviews. A pilot interview was conducted with two volunteers to test the interview  guide for its ease of understanding, cultural appropriateness and ability to capture the relevant information. |
| 18 | **Repeat interviews** | Not stated | Not stated | Not stated | Not conducted | Not conducted | Not conducted |
| 19 | **Audio/visual recording** | Audio-recording | Audio-recording | Audio-recording | Audio-recorded | Audio-recording | Audio-recording |
| 20 | **Field notes** | Field notes were not provided | Field notes were provided | Field notes were provided | Field notes were not provided | Field notes were provided | Field notes were provided |
| 21 | **Duration** |  | 2 hours | 17 and 90 min | Not stated | 25-90 minutes | 25-90 minutes |
| 22 | **Data saturation** | Not stated | Not stated | Data saturation was achieved | Not stated | Data saturation was achieved | Data saturation was achieved |
| 23 | **Transcripts returned** | Transcripts were not returned to participants | Transcripts were not returned to participants | Transcripts were not returned to participants | Not stated | Transcripts were not returned to participants | Transcripts were not returned to participants |
| 24 | **Number of data coders** | Not stated | 3 | Not stated | Not stated | 5 | 5 |
| 25 | **Description of the coding tree** | Authors provided a description of the coding tree | Authors provided a description of the coding tree | Authors provided a description of the coding tree | Yes | Authors provided a description of the coding tree | Authors provided a description of the coding tree |
| 26 | **Derivation of themes** | Themes were derived from data | Themes were derived from data | Themes were derived from data | Themes were derived from data | Themes were derived directly from the data following the coding process. | Themes were derived directly from the data following the coding process. |
| 27 | **Software** | MAXQDA 12 | NVivo 11 software | Not stated | Not stated | NVivo 11 software | NVivo 11 software |
| 28 | **Participant checking** | Not stated | Not stated | Not stated | Not stated | The researcher in the current study conducted a quasi-member checking simultaneously at the process of data collection. | The researcher in the current study conducted a quasi-member checking simultaneously at the process of data collection. |
| 29 | **Quotations presented** | Yes | Yes | Yes | Yes | Yes | Yes |
| 30 | **Data and findings consistent** | Yes | Yes | Yes | Yes | Yes | Yes |
| 31 | **Clarity of major themes** | Yes | Yes | Yes | Yes | Yes | Yes |
| 32 | **Clarity of minor themes** | Yes | Yes | Yes | Yes | Yes | Yes |

|  | **Checklist** | **[40]**  **Noakes (2010)** | **[41]**  **Nortved et al, 2016** | **[42]**  **Nyaaba et al., 2019** | **[43]**  **Omar et al, 2017** | **[44]**  **Renedo et al, 2019** | **[45]**  **Said et al., 2021** |
| --- | --- | --- | --- | --- | --- | --- | --- |
| 1 | **Interviewer/facilitator identified** | HN | Not stated | Not stated | Not stated | AR | Not stated |
| 2 | **Credentials** | No | Not stated | Not stated | Not stated | Not stated | Not stated |
| 3 | **Occupation** | Diabetes Specialist Nurse | Not stated | Not stated | Not stated | Yes | Not stated |
| 4 | **Gender** | Female | Not clear | Male, female | Male | Male and Female | Male |
| 5 | **Experience and training** | No | Not stated | Not stated | Yes | Not stated | Not stated |
| 6 | **Relationship established** | No | Not stated | Not stated | Not stated | Yes | Not stated |
| 7 | **Participant knowledge of the interviewer** | No | Not stated | Not stated | Not stated | No | Not stated |
| 8 | **Interviewer characteristics** | No | Not stated | Not stated | Not stated | Yes | Not stated |
| 9 | **Methodological orientation and Theory** | Thematic content analysis. | Not clear | Thematic | Thematic analysis | Grounded Theory and thematic analysis | Not stated |
| 10 | **Sampling** | Purposive sampling | Not stated | Purposive | Not stated | Not stated | Purposive and snowball sampling |
| 11 | **Method of approach** | Face-to-face | Face-to-face | Face-to-face | Face-to-face | Face-to-face | Face-to-face |
| 12 | **Sample size** | 13 | 14 | 55 | 36 | 48 | 31 |
| 13 | **Non-participation** | None | 3 | Not stated | Not stated | 16, they did not return phone calls or were too busy | Not stated |
| 14 | **Setting of data collection** | South London NHS Hospital Trust | Rehabilitation hospital; outpatient clinic | Community | Community | Communities, hospitals | Community |
| 15 | **Presence of nonparticipants** | No | Not stated | Not stated | Not stated | Not stated | Not stated |
| 16 | **Description of sample** | N: *5 African women* (Total -13)  Gender: 5m, 8w  Age: 44-77  Location: London  Setting: South London NHS Hospital Trust | N: *4 from African countries* (Total - 14)  Gender: 14w  Age: 30-56  Location: South Norway  Setting: Rehabilitation hospital; outpatient clinic | N: *20 based in Amsterdam* (Total- 55)  Gender: 14w, 41m  Age: 30-73  Location: Amsterdam, Kumasi, Tamale, Bolgatanga  Setting: community | N: 36  Gender: 36m  Age: 18 - 60  Location: Melbourne  Setting: community | N=48*  Gender: 30w, 18m  Age: 13 – 21  Location: London; ‘one other English city’  Setting: Communities, hospitals | N=31  Gender: 31w  Age: 18 - 34  Location: Melbourne  Setting: community |
| 17 | **Interview guide** | No | Yes | Interview guide was provided | Interview guide was provided | Not provided | Not provided |
| 18 | **Repeat interviews** | Not conducted | Not stated | Not provided | Yes | Yes | Not stated |
| 19 | **Audio/visual recording** | Audio-recording | Audio-recording | Not stated | Audio-recorded | Yes | Not stated |
| 20 | **Field notes** | Field notes were provided | Field notes were provided | Field notes were not provided | Field notes were not provided | Field notes were not provided | Field notes were not provided |
| 21 | **Duration** | I Hour | 35 and 110 min | 30-77mins | 90–120 minutes. | 60-90 mins | Not stated |
| 22 | **Data saturation** | Data saturation was not achieved | Not stated | Not stated | Not stated | Yes | Not stated |
| 23 | **Transcripts returned** | Transcripts were not returned to participants | Transcripts were not returned to participants | Not stated | Yes | No | Not stated |
| 24 | **Number of data coders** | 1 | Not stated | 3 | 2 | Not stated | Not stated |
| 25 | **Description of the coding tree** | Authors provided a description of the coding tree | Authors provided a description of the coding tree | Yes | Yes | Yes | Yes |
| 26 | **Derivation of themes** | Themes were derived from data | Themes were derived from data | Themes were derived from data | Themes were derived from data | Yes | Not stated |
| 27 | **Software** | No | Not stated | Nvivo | Not stated | No | Not stated |
| 28 | **Participant checking** | No | Not stated | Not stated | Not stated | No | Not stated |
| 29 | **Quotations presented** | Yes | Yes | Yes | Yes | Yes | Yes |
| 30 | **Data and findings consistent** | Yes | Yes | Yes | Yes | Yes | Yes |
| 31 | **Clarity of major themes** | Yes | Yes | Yes | Yes | Yes | Yes |
| 32 | **Clarity of minor themes** | Yes | Yes | Yes | Yes | Not clear | Yes |

|  | **Checklist** | **[46]**  **Sauvegrain et al., 2017** | **[47]**  **Secker & Harding (2002).** | **[48]**  **Secker (2002).** | **[49]**  **Siad et al 2018** | **[50]**  **Smith et al., 2019** | **[51]**  **Wagstaff et al, 2018** |
| --- | --- | --- | --- | --- | --- | --- | --- |
| 1 | **Interviewer/facilitator identified** | Not stated | Not stated | Not stated | Not stated | Not stated | Not stated |
| 2 | **Credentials** | Not stated | Yes | Yes | Yes | Not stated | Yes |
| 3 | **Occupation** | Not stated | Not stated | Not stated | Not stated | Not stated | Not stated |
| 4 | **Gender** | Female | Not stated | Not stated | Not stated | Male, Female | Not stated |
| 5 | **Experience and training** | Not stated | No stated | Not stated | Not stated | Not stated | Not stated |
| 6 | **Relationship established** | Yes | Not stated | No | Not stated | Not stated | No |
| 7 | **Participant knowledge of the interviewer** | Yes | No | Not stated | Not stated | Not stated | No |
| 8 | **Interviewer characteristics** | Not stated | Not stated | Not stated | Not stated | Not stated | Not stated |
| 9 | **Methodological orientation and Theory** | Thematic | Not stated | Not stated | inductive thematic content analysis | Thematic | Interpretative Phenomenological Analysis |
| 10 | **Sampling** | Not stated | No | No | Not stated | Not stated | purposive sampling |
| 11 | **Method of approach** | Face-to-face | Face-to-face | Face-to-face | Face-to-face | Face-to-face | Face-to-face |
| 12 | **Sample size** | 33 | 26 | 26 | 10 | 31 | 7 |
| 13 | **Non-participation** | Not stated | None | None | 9 | Not stated | 7 declined or did not participate in the final interviews |
| 14 | **Setting of data collection** | Three public maternity units | Mental health resource centre | Mental health resource centre | Diabetes-in-pregnancy (DIP) clinics | Community | Community |
| 15 | **Presence of nonparticipants** | Hospital | No | Not stated | Not stated | Not stated | No |
| 16 | **Description of sample** | N: 33  Gender: 33w  Age: 22-45  Location: Paris  Setting: Three public maternity units | N: *6 of African heritage* (Total – 26)  Gender: 10m, 16w  Age: 18 - 64  Location: London  Setting: Mental health resource centre | N: *6 of African heritage* (Total – 26)  Gender: 10m, 16w  Age:18 – 64  Location: London  Setting: Mental health resource centre | N: 10  Gender: 10w  Age:18+  Location: Calgary  Setting: Diabetes-in-pregnancy (DIP) clinics | N: *2 from Africa?** (Total - 24)  Gender: m/w  Age: ‘adults and youth’  Location : Launceston, Australia  Setting: community | N=7*  Gender: 7m  Age: 31 – 64  Location: West Midlands:  Setting: community |
| 17 | **Interview guide** | Not provided | Not provided | No | Not stated | Not stated | No |
| 18 | **Repeat interviews** | Not provided | Not provided | Not conducted | Not stated | Not stated | Yes |
| 19 | **Audio/visual recording** | Not stated | Yes | Yes | Yes | Audio | Yes |
| 20 | **Field notes** | Not stated | Field notes were not provided | Field notes were not provided | Field notes were provided | Not stated | Field notes were provided |
| 21 | **Duration** | Not stated | I hour | 1 hour | Not stated | Not stated | Not stated |
| 22 | **Data saturation** | 35-150 mins | Not stated | Not stated | Yes | Not stated | Not stated |
| 23 | **Transcripts returned** | Not stated | No | No | Not stated | Not stated | No |
| 24 | **Number of data coders** | Not stated | Not stated | Not stated | Not stated | 2 | Not stated |
| 25 | **Description of the coding tree** | 1 | Not stated | Not stated | Yes | Yes | Yes |
| 26 | **Derivation of themes** | Not stated | Yes | Yes | Themes were derived from data | Themes were derived from data | Yes |
| 27 | **Software** | Not stated | WINMAX | WINMAX program | NVivo v.10.2 | Nvivo | Not stated |
| 28 | **Participant checking** | Nvivo | No | No | Not stated | Not stated | Yes |
| 29 | **Quotations presented** | Yes | Yes | Yes | Yes | Yes | Yes |
| 30 | **Data and findings consistent** | Yes | Yes | Yes | Yes | Yes | Yes |
| 31 | **Clarity of major themes** | Yes | Yes | Yes | Yes | Yes | Yes |
| 32 | **Clarity of minor themes** | Yes | No | No | Yes | Yes | No |

|  | **Checklist** | **[52]**  **Wallin and Ahlstrom 2010** | **[53]**  **Wallin et al 2007** | **[54]**  **Weich et al 2012** |
| --- | --- | --- | --- | --- |
| 1 | **Interviewer/facilitator identified** | Not stated | Not stated | Not stated |
| 2 | **Credentials** | Yes | Yes | Not stated |
| 3 | **Occupation** | Not stated | Yes | Not stated |
| 4 | **Gender** | Female and male | 2x females and male | Not stated |
| 5 | **Experience and training** | Not stated | Not stated | Not stated |
| 6 | **Relationship established** | Not stated | Not stated | Yes |
| 7 | **Participant knowledge of the interviewer** | Not stated | Not stated | No |
| 8 | **Interviewer characteristics** | Not stated | Not stated | Yes |
| 9 | **Methodological orientation and Theory** | Content analysis | Content analysis | Not stated |
| 10 | **Sampling** | Not stated | Not stated | case sampling |
| 11 | **Method of approach** | Face-to-face | Telephone calls | Face-to-face |
| 12 | **Sample size** | 19 | 19 | 40 were interviewed (22 males, 18 females) |
| 13 | **Non-participation** | 14 | 14 | 70, mainly due to lack of time |
| 14 | **Setting of data collection** | Primary health centres | Primary health centres, Public places and work place | PCT |
| 15 | **Presence of nonparticipants** | Not stated | Not stated | Not stated |
| 16 | **Description of sample** | N: 19  Gender: 8m, 11w  Age: 30 - 83  Location: Sweden  Setting: Primary health centres | N: 19  Gender: 8m, 11w  Age: 30 - 83  Location: Sweden  Setting: Primary health centres, Public places and work place | N= *1 African man* (total – 40)  Gender: 22m, 18w  Age: 18-65  Location: Birmingham  Setting: PCT |
| 17 | **Interview guide** | Yes | Not stated | Yes |
| 18 | **Repeat interviews** | Not stated | Not stated | Not conducted |
| 19 | **Audio/visual recording** | Field notes were provided | Field notes were provided | Field notes were provided |
| 20 | **Field notes** | Yes | Yes | Yes |
| 21 | **Duration** | 2 hours | 2 hours | 20 min to 2 h, averaging 1 hour |
| 22 | **Data saturation** | Not stated | Not stated | Yes |
| 23 | **Transcripts returned** | Not stated | Not stated | No |
| 24 | **Number of data coders** | 2 | 3 | 3 |
| 25 | **Description of the coding tree** | Content analysis | Yes | Yes |
| 26 | **Derivation of themes** | Themes were derived from data | Themes derived from data | Themes derived from data |
| 27 | **Software** | Not stated | Not stated | NVivo 8.0 |
| 28 | **Participant checking** | Not stated | Not stated | No |
| 29 | **Quotations presented** | Yes | Yes | Yes |
| 30 | **Data and findings consistent** | Yes | Yes | Yes |
| 31 | **Clarity of major themes** | Yes | Yes | Yes |
| 32 | **Clarity of minor themes** | Yes | Yes | No |
